# Rig-G is a growth inhibitory factor of lung cancer cells that suppresses STAT3 and NF-κB

**DOI:** 10.18632/oncotarget.11797

**Published:** 2016-09-01

**Authors:** Dong Li, Junjun Sun, Wenfang Liu, Xuan Wang, Robert Bals, Junlu Wu, Wenqiang Quan, Yiwen Yao, Yu Zhang, Hong Zhou, Kaiyin Wu

**Affiliations:** ^1^ Department of Clinical Laboratory, Shanghai Tongji Hospital, Tongji University School of Medicine, 200065 Shanghai, China; ^2^ Department of General Surgery, Shanghai Tongji Hospital, Tongji University School of Medicine, 200065 Shanghai, China; ^3^ Department of Pharmacy, Putuo People's Hospital, 200060 Shanghai, China; ^4^ Department of Internal Medicine V – Pulmonology, Allergology, Respiratory Intensive Care Medicine, Saarland University Hospital, 66424 Homburg, Germany; ^5^ Institute of Pathology, Charité Medical University, 10117 Berlin, Germany

**Keywords:** Rig-G, lung cancer, growth inhibition, NF-κB, STAT3

## Abstract

The expression of the retinoic acid-induced G (Rig-G) gene, an all trans retinoic acid (ATRA)-inducible gene, was observed in multiple cancer cells, including lung cancer cells. However, whether Rig-G is a tumor suppressor in lung cancer is unknown. Here, we found that ectopic expression of Rig-G can lead to a significant decrease in proliferation of lung cancer cells, resulting in an inhibition of tumor growth. Rig-G knockdown results in a modest increase in cell proliferation, as well as confers an increase in colony formation. Furthermore, transcriptome and pathway analyses of cancer cells revealed a fundamental impact of Rig-G on various growth signaling pathways, including the NF-κB pathway. Rig-G inhibits NF-κB activity by suppressing STAT3 in lung cancer cells. The downregulation of miR21 and miR181b-1 and subsequent activation of PTEN/Akt and CYLD/IκB signaling axis leading to decreased NF-κB activity required to maintain the tumor-inhibiting effect of Rig-G.. Our findings contribute to a better understanding of the antitumor effect mechanism of Rig-G, as well as offer a novel strategy for lung cancer therapy.

## INTRODUCTION

Lung cancer currently remains the leading cause of death from cancer in the world [1]. Among lung cancers, non–small cell lung cancer (NSCLC) constitutes 80% of all lung cancers [2,3]. Despite advances in early detection and standard treatment, lung cancer is often diagnosed at an advanced stage and has poor prognosis [4]. Therefore, prevention and treatment of lung cancer are the focus of intensive current research [5].

Retinoic acid-induced gene G (Rig-G) is an all trans retinoic acid (ATRA)-inducible gene, previously identified from an acute promyelocytic leukemia (APL) cell line NB4 by using the technique of differential Display PCR [6,7]. Rig-G gene expression can be induced not only in NB4 cells but also in various types of solid carcinoma cells, including head and neck squamous carcinoma cells, NSCLC H460 and A549 cells, cervical carcinoma HeLa cells, and epithelium-like WISH cells [8]. Studies have shown that Rig-G is a growth inhibitor for leukemia cells, whose anti-proliferative effect was induced via the upregulation of p21 and p27, two negative regulators of cell cycle progression [8]. Rig-G blocks nuclear export of p27 and its subsequent proteolysis by interfering with the normal function of JAB1, a co-activator of the AP1 transcription factor. This event then mediates the nuclear export of p27 and facilitates p27 to degrade via ubiquitin/proteasome pathway in cytoplasm, finally increasing p27 protein levels and inhibiting cell growth [8]. Rig-G also upregulates p21 at the transcriptional level by decreasing c-Myc expression, thereby inhibiting cell proliferation and facilitating cell differentiation [8].

NF-κB and STAT3 play pivotal roles in various aspects of the tumorigenic process in several cancer entities, including colon, lung cancer, and hepatocellular carcinoma [9–11]. These are powerful activators of malignancy and are pre-requisites for the expression of a variety of target genes that are important for cell proliferation, survival, angiogenesis, invasion, and metastasis [12,13]. On the contrary, in immune cells, NF-κB and STAT3 control the expression of other cytokines and inflammatory/immune mediators that mediate NF-κB and STAT3 activation in cancer cells, including IL-1, IL-6, and TNFα [14]. The interaction of NF-κB and STAT3 has been found in some human cancers, including colon, gastric, and liver [12,15,16]. NF-κB physically interacts with STAT3, which may result in either specific transcriptional synergy or repression of NF-κB/STAT3 regulated genes [12,16]. STAT3 may interact with RelA/p65 in the nucleus and prolong the presence of active NF-κB in the nucleus [16]. STAT3 activation of miR-21 and miR-181b-1 has dramatic effects on NF-κB activity in the tumor cells [17]. It was also observed that STAT3 inactivation may be responsible for decreased NF-κB activity [17]. Despite these versatile interactions, NF-κB and STAT3 cooperate to promote the development and progression of tumors [15,16].

The aim of the present study was to determine whether overexpression of Rig-G in lung cancer cells alters its growth and which growth inhibition signaling pathways in cancer cells are involved. Here we use an inducible Rig-G expression model to study the effects of Rig-G in the lung cancer cell, and identify the underlying molecular mechanisms.

## RESULTS

### ATRA upregulates Rig-G expression and inhibits the growth of lung cancer cells *in vitro*

**Figure 1:**
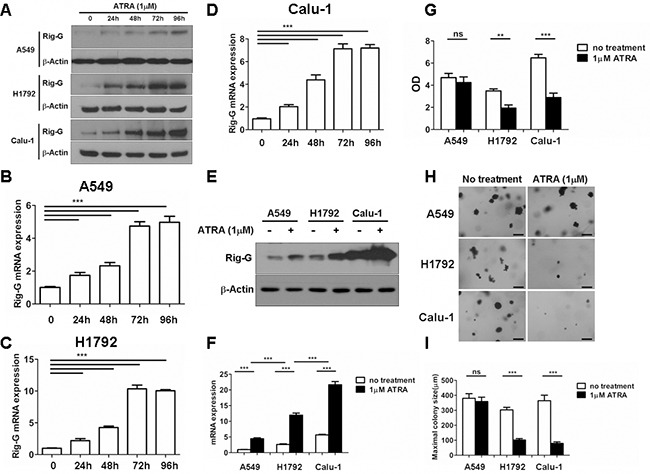
ATRA upregulates Rig-G expression and inhibits the growth of lung cancer cells *in vitro* **A–F.** Total protein and RNA from A549, H1792, and Calu-1 cells were isolated after treatment with 1 μM of ATRA for 96 h. The expression of Rig-G protein was analyzed by western blot (A and E). The expression of Rig-G mRNA in three NSCLC cell lines (B–D and F) was measured by real-time PCR. The results are expressed as the mean ± SEM, ***p < 0.001 **G.** Lung cancer cell lines A549, H1792, and Calu-1 were treated with 1 μm ATRA for 96 h, after which cell proliferation was measured by ELISA (BrdU labeling) analysis. The results are expressed as the mean ± SEM, **p < 0.01; ***p < 0.001; n.s., not significant. **H.** A549, H1792, and Calu-1 cells were treated with 1 μm ATRA and assessed for growth in soft agar by using the anchorage-independent colony formation assay. Scale bars = 500 μm. **I.** The maximum colony size of A549, H1792, and Calu-1 cells in a soft-agar assay was determined. The results are expressed as the mean ± SEM, ***p < 0.001; n.s., not significant.

In our previous work, we showed that Rig-G, whose expression is triggered by ATRA, plays an important role in leukemia cell growth inhibition [8]. To assess whether ATRA-induced Rig-G induction and cell growth inhibition are relevant in lung cancer cells, three NSCLC cell lines were used to detect the induction of Rig-G at the protein and mRNA levels. A previous report showed that the peak plasma levels of a single oral dose of 45 mg/m^2^ that is administered to patients is 1 μM ATRA; therefore, 1 μM ATRA was used in the present study [18]. Western blot analysis showed that treatment with 1 μM ATRA for 96 h significantly increased the expression of Rig-G in A549, H1792, and Calu-1 cells (Figure 1A). The levels of Rig-G mRNA were perfectly consistent with the kinetics of Rig-G protein expression (Figure 1B, 1C, 1D). Interestingly, Rig-G expression in A549 cells was significantly lower than that in Calu-1 and H1792 cells (Figure 1E, 1F). The cell lines were treated for 96 h and a BrdU ELISA cell proliferation assay was subsequently performed. Growth inhibition of Calu-1 and H1792 cells significantly increased after treatment with ATRA, whereas A549 cells were less sensitive to ATRA (Figure 1G). An anchorage-independent colony formation assay was conducted to determine whether ATRA reduces the ability of soft agar colony formation in Calu-1, A549, and H1792 cells. ATRA significantly inhibited the ability of Calu-1 and H1792 cells to grow on soft agar, whereas minimal inhibition was observed after ATRA treatment of A549 cells (Figure 1H, 1I). These results were in agreement to those obtained by measuring cell proliferation. Collectively, these results indicate that ATRA stimulates Rig-G expression and inhibits lung cancer cell growth in a cell-dependent manner.

### Rig-G inhibits lung cancer cell growth and impairs tumor development in xenograft models

**Figure 2:**
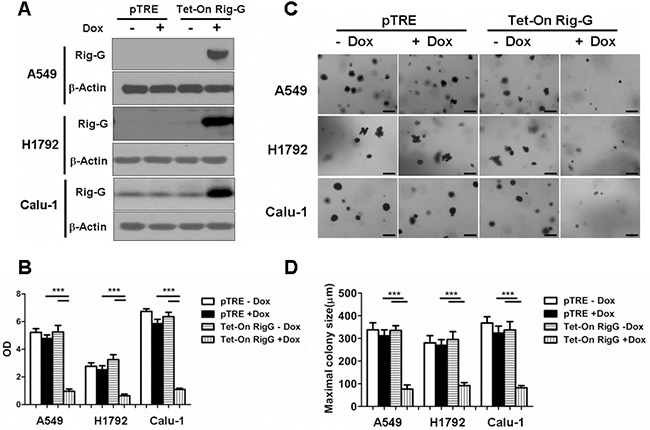
Rig-G inhibits lung cancer cell growth **A.** The lung cancer cell (A549, H1792, and Calu-1) sublines pTRE and Tet-on Rig-G were cultured, respectively, in the presence or absence of Dox (2μg/mL) for 24h. The expression of Rig-G protein was detected by immunoblotting. **B.** The proliferation of the indicated cells was measured by ELISA (BrdU labeling) analysis. The results are expressed as the mean ± SEM, ***p < 0.001. **C.** The growth of the indicated cells in soft agar was assessed by using an anchorage-independent colony formation assay. Scale bars = 500 μm. **D.** The maximum colony size of A549, H1792, and Calu-1 cells in a soft-agar assay was determined. The results are expressed as the mean ± SEM, ***p < 0.001.

**Figure 3:**
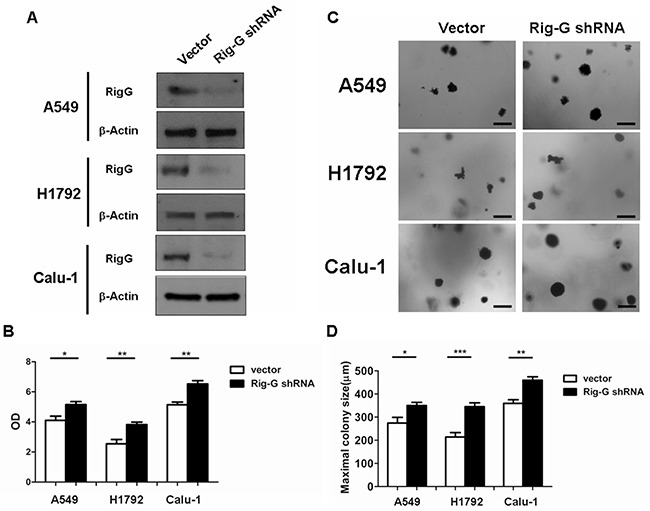
Rig-G knockdown increases lung cancer cell growth **A.** Lung cancer cell A549, H1792, and Calu-1 were transduced with indicated shRNA. After selection, expression of Rig-G proteins was analysed. **B.** The indicated cells were transduced with control or Rig-G shRNA plasmids, and their proliferation was measured by ELISA (BrdU labeling) analysis. The results are expressed as the mean ± SEM, *p < 0.05; **p < 0.01. **C.** The growth of tumor cells transduced with indicated shRNA plasmids in soft agar was assessed by using an anchorage-independent colony formation assay. Scale bars = 500 μm. **D.** The maximum colony size assay in A549, H1792, and Calu-1 cells transfected with the indicated shRNA plasmids. The results are expressed as the mean ± SEM, *p < 0.05; **p < 0.01; ***p < 0.001.

**Figure 4:**
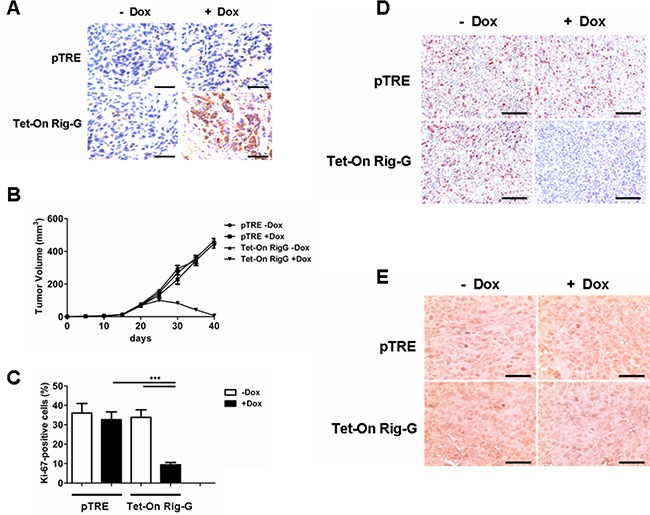
Rig-G protein results in decreased tumor growth *in vivo* **A.** Nude mice were injected subcutaneously with the indicated cell lines, and the expression of Rig-G was examined by immunohistochemistry of paraffin-embedded sections. Scale bars = 100 μm. **B.** Tumors overexpressing Rig-G showed significantly slower growth (n = 5). **C.** Percentage of Ki-67-positive tumor cells. The results are expressed as the mean ± SEM, (n = 5), ***p< 0.001. **D.** Paraffin-embedded tumor sections were analyzed after staining with an anti-Ki-67 antibody. Scale bars = 100 μm. **E.** Apoptotic cells were identified by TUNEL staining. Scale bars = 100 μm.

As ATRA treatment results in a significant increase in Rig-G expression and inhibition of lung cancer cell growth, we hypothesized that Rig-G induces growth inhibition of lung cancer cells. In accord with this idea, Calu-1, A549, and H1792 cells stably expressing Rig-G were generated using the Tet-On expression system. In the presence of doxycycline (Dox), the induced expression of Rig-G in Tet-On Rig-G stably expressing cell lines was confirmed by western blot analysis (Figure 2A). Empty vector pTRE was used as control. The upregulation of Rig-G in Tet-On Rig-G stably expressing cell lines resulted in a very low background of Rig-G expression in control cells (Figure 2A). As expected, the overexpression of Rig-G in Calu-1 and H1792 cells resulted in a significant inhibition of cell growth after the addition of Dox (Figure 2B). Analysis of anchorage-independent colony formation further showed that cellular expression of Rig-G significantly decreased the ability of lung cancer cell lines Calu-1 and H1792 to grow on soft agar (Figure 2C, 2D). Strikingly, A549 cells, which are resistant to ATRA, overexpressed Rig-G that strongly inhibited cell growth as well as the ability to form colonies in soft agar (Figure 2B, 2C, 2D). In addition, we also examined whether the loss of Rig-G affected the growth of lung tumor cells. We inhibited Rig-G expression by transfection with Rig-G shRNA in tumor cells (Figure 3A). In three cell lines, inhibition of Rig-G results in a modest increase in cell proliferation, as well as confers an increase in colony formation (Figure 3B, 3C, 3D). Nude mice were injected subcutaneously with A549 cells carrying a regulated Rig-G expression cassette or control cassette to generate tumors. Tumor-bearing animals were fed Dox or water to regulate Rig-G expression in mice xenografts (Figure 4A). Expression of Rig-G significantly suppressed tumor growth, as a reduction in tumor size was observed relative to that in the control (Figure 4B). The proliferation of tumor cells was then examined via immunohistochemical staining for Ki-67. Consistent with the observed changes in the xenografts, the number of cells expressing Ki-67 was significantly lower in tumors from mice showing Rig-G overexpression compared to mice showing a low level of Rig-G expression (Figure 4C, 4D). Next, to investigate whether Rig-G has an impact on apoptosis in tumor cells, apoptotic cells were identified by *in situ* terminal-transferase dUTP-mediated nick end-labeling (TUNEL) assay, which showed no significant differences between groups (Figure 4E). These results indicate that Rig-G appears to play a critical role in the inhibition of lung tumor growth, most likely through inhibiting the proliferation of malignant cells, without affecting rate of apoptosis.

### Rig-G inhibits a proliferation program and NF-κB activation in cancer cells

**Figure 5:**
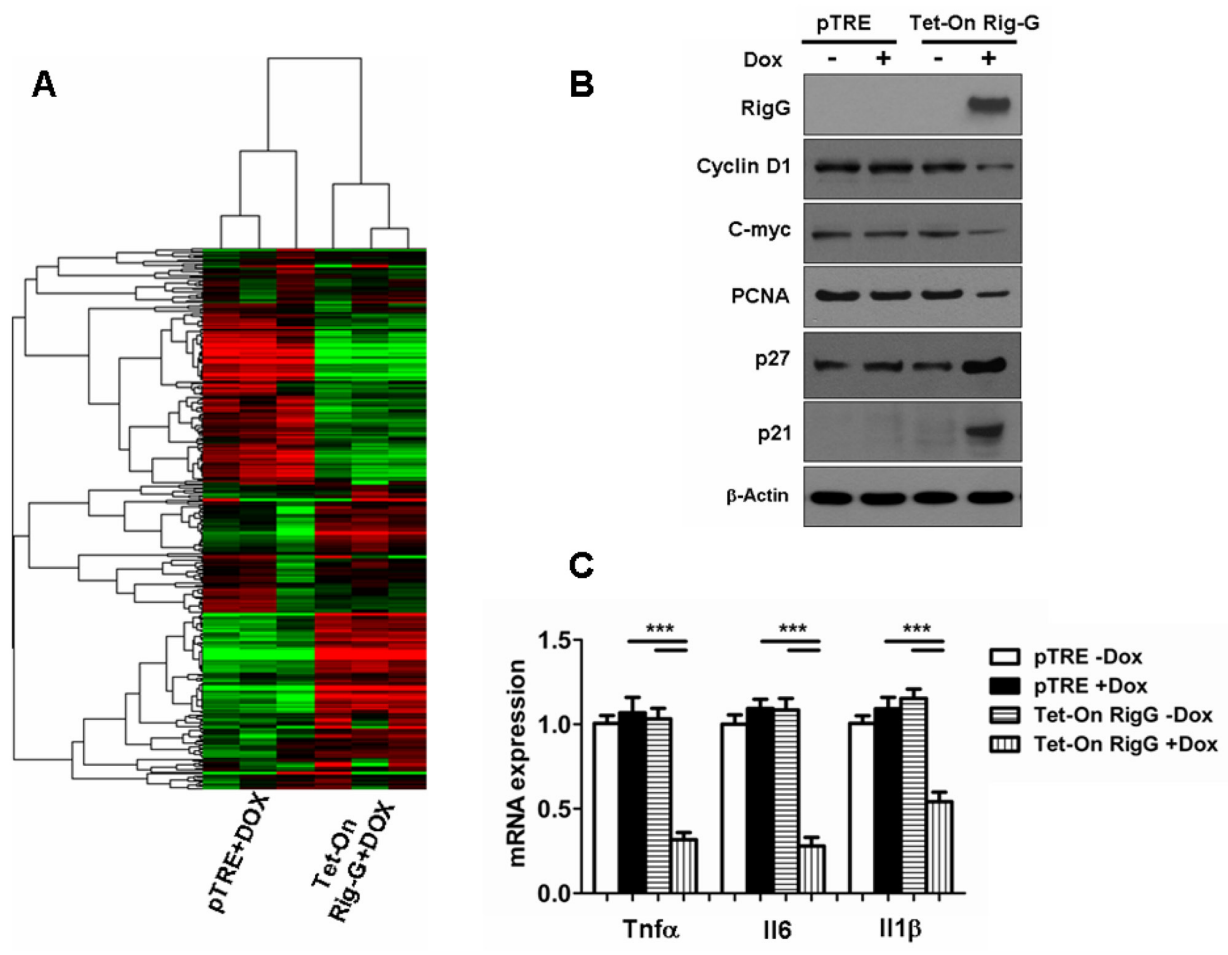
Rig-G inhibits specific growth pathways and inflammatory responses in tumor cells **A.** Heat map of the experimental groups. Dendrogram and cluster analyses show a differential gene expression profile pattern in A549 cell lines with (right) and without (left) Rig-G overexpression. Green indicates downregulation, and red indicates upregulation. **B.** The expression of proliferation-related proteins in the indicated cells was analyzed by western blotting. **C.** Induction of inflammatory cytokine mRNAs in the indicated cells was measured by real-time PCR. The results are expressed as the mean ± SEM (n = 5), ***p < 0.001.

Because of the strong effect of Rig-G in inhibiting growth and colony formation in the ATRA-resistant cell line A549 cell, subsequent experiments were performed using the A549 cells. To characterize differentially expressed mRNA during tumor growth inhibition, we measured the mRNA profile of A549 cells with and without Rig-G overexpression. A549 cells without Rig-G overexpression (pTRE+DOX) were used as control, and A549 cells with Rig-G overexpression (Tet-On Rig-G+DOX) were used as the experimental group. Microarray analysis showed that Rig-G overexpression results in the downregulation of the Kyoto Encyclopedia of Genes and Genomes (KEGG) cancer, cell cycle, NF-κB pathways [Gene Set Enrichment Analysis (GSEA): P = 0.017, 0.001, and 0.001] and other pathways and categories related to tumorigenesis (Table 1). Notably, KEGG chemokine and cytokine-cytokine receptor interactions, which are responsible for generating an inflammatory response, were also downregulated by Rig-G overexpression. Next, we generated a heat map that shows a differentially gene expression profile pattern between with and without Rig-G overexpression in A549 cells (Figure 5A).

**Table 1:** 20 downregulated pathways found by a GSEA between A549 cell with and without Rig-G overexpression

KEGG Pathway	P value	Enrichment
Pathways in cancer	0.017	Down
Wnt signaling pathway	0.006	Down
NF-κB signaling pathway	0.001	Down
Axon guidance	0.023	Down
Renal cell carcinoma	0	Down
Cell cycle	0.001	Down
Chemokine signaling pathway	0	Down
Cytokine-cytokine receptor interaction	0	Down
Arginine and proline metabolism	0.007	Down
Retinol metabolism	0.002	Down
Complement and coagulation cascades	0.003	Down
VEGF signaling pathway	0.002	Down
Terpenoid backbone biosynthesis	0	Down
Glycerophospholipid metabolism	0.031	Down
Aminoacyl-tRNA biosynthesis	0.018	Down
Tryptophan metabolism	0.001	Down
Cell adhesion molecules (CAMs)	0.0004	Down
DNA replication	0.002	Down
Endocytosis	0.004	Down
Nucleotide excision repair	0	Down

The enrichment is relative to the enrichment specifier and relative to the A549 without Rig-G overexpression.

We then investigated whether specific growth pathways and inflammatory response are inhibited by Rig-G. In contrast to the A549 cells without Rig-G overexpression, expression of cyclin D1, c-myc, and PCNA was inhibited in response to Rig-G overexpression in A549 cells (Figure 5B). We also observed that the expression of cell cycle inhibitors p21 and p27 was high in A549 cells with Rig-G overexpression and low in A549 cells without Rig-G overexpression (Figure 5B). These results were in correlation with those obtained by measuring xenograft tumor size or Ki-67 immunostaining. Furthermore, inflammation-related genes were examined by real-time PCR, which showed that the expression of Il6, Tnfα, and Il1β were strongly inhibited in Rig-G overexpressing cells (Figure 5C). These findings indicate that Rig-G mediates the induction of inflammatory mediators, thereby suggesting that the cell growth suppressive actions of Rig-G are also likely to be correlated with the modulation of inflammatory genes.

**Figure 6:**
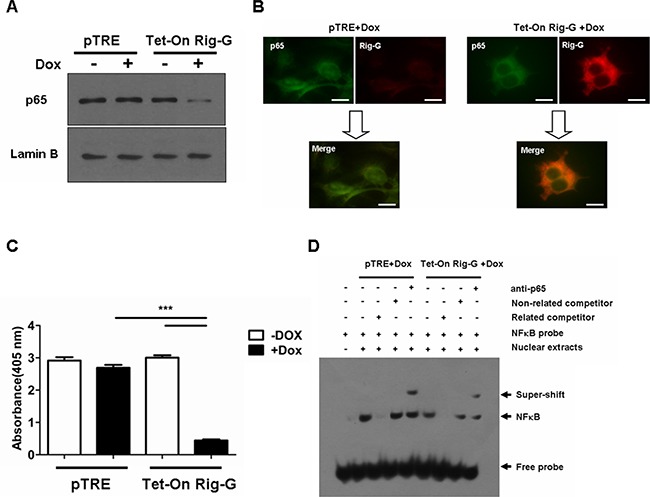
Rig-G inhibits NF-κB activation in A549 cells **A.** The A549 sublines pTRE and Tet-On Rig-G were cultured, respectively, in the absence or presence of Dox (2 μg/mL) for 24 h. Nuclear protein from A549 cells was isolated. The expression of the indicated protein was analyzed by Western blot. **B.** Double-color immunofluorescence analysis was performed with the combination of anti- Rig-G (red) and anti-p65 (green). Scale bars = 10 μm. **C.** NF-κB activation was measured by ActivELISA assay in A549 cells with and without Rig-G overexpression. The results are expressed as the mean ± SEM (n = 3), ***p < 0.001. **D.** EMSA analysis of the DNA binding of NF-κB.

NF-κB has a key role in the oncogenesis, apoptosis, immune, and inflammatory responses that modulate transcription of various genes that encode growth factors, anti-apoptotic proteins, cytokines, and cell adhesion molecules [19]. We examined whether Rig-G inhibited growth and the inflammatory response of A549 cells through a NF-κB intermediate. Western blotting was performed on cell nuclear proteins to assess the levels of p65 in A549 cells with and without Rig-G overexpression. The level of p65 in nuclear proteins was significantly lower in Rig-G overexpression cells compared with the cells without Rig-G overexpression (Figure 6A). By immunofluorescence staining, we noted that when Rig-G overexpression in A549 cells, the nuclear p65 was markedly diminished. A colocalization of p65 and Rig-G in the cytoplasm was shown by superimposition of the staining for these two proteins, indicating that decreased p65 level in nuclear of A549 with Rig-G overexpression is due to the interference of Rig-G (Figure 6B). Furthermore, NF-κB activation was measured with a TransAM® ELISA kits, and found that Rig-G resulted in a significant inhibition of NF-κB activation in A549 cells (24 h after Dox treatment) (Figure 6C). This result was confirmed by EMSA determination. Cell nuclear proteins were used to test for their binding to NF-κB probe. By EMSA, we observed a decrease in NF-κB DNA binding activity in A549 cells with Rig-G overexpression (Figure 6D). These results indicated that Rig-G is an important NF-κB inhibitor in NSCLC cells.

**Figure 7:**
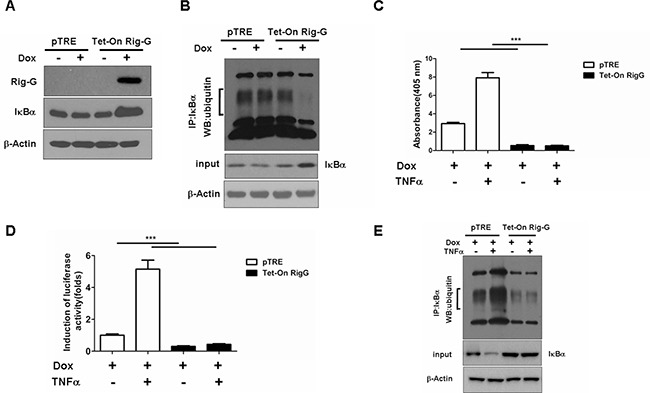
Effects of Rig-G protein on IκBα degradation **A.** Western blotting analysis of IκBα expression in A549 sublines pTRE and Tet-On Rig-G. The cells were cultured respectively in the presence or absence of Dox (2 μg/mL) for 24 h. **B.** The pTRE and Tet-On Rig-G cells were incubated with the proteasome inhibitor MG132 (20μM) for 5h, lysed, and subjected to immunoprecipitation (IP) with anti- IκBα antibody. The resulting precipitates were detected by Western blot analysis with antiubiquitin antibodies. **C.** NF-κB activation in the indicated cells untreated or treated with 1 ng/mL TNFα for 1 h. The results are expressed as the mean ± SEM (n = 3), ***p < 0.001. **D.** The effect of Rig-G on the TNF-mediated inducibility of an NF-κB reporter construct. **E.** The indicated cells were incubated with the proteasome inhibitor MG132 (20μM) for 5h, and cells were untreated or treated with 1 ng/mL TNFα for 1 h, and cell lysates were immunoprecipitated (IP) with an anti-IκBα antibody, followed by Western blot analysis with an anti-ubiquitin antibody.

**Figure 8:**
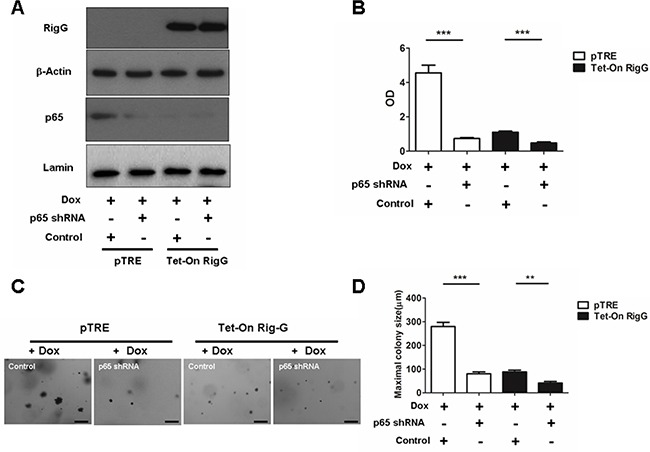
NF-κB is important in RIG-G-induced cell growth inhibition **A.** A549 sublines pTRE and Tet-On Rig-G were transduced with indicated shRNA plasmids. After selection, the cells were cultured, respectively, in the absence or presence of Dox (2 μg/mL) for 24 h, expression of the indicated proteins was analysed. **B.** The indicated cells were transduced with control or p65 shRNA plasmids, and their proliferation was measured by ELISA (BrdU labeling) analysis. The results are expressed as the mean ± SEM, ***p < 0.001. **C.** The growth of tumor cells transduced with indicated shRNA plasmids in soft agar was assessed by using an anchorage-independent colony formation assay. Scale bars = 500 μm. **D.** The maximum colony size assay in A549 sublines pTRE and Tet-On Rig-G cells transfected with the indicated shRNA plasmids. The results are expressed as the mean ± SEM, **p < 0.01; ***p < 0.001.

We observed that IκBα, a NF-κB inhibitory protein, was strongly upregulated in Rig-G overexpression cells (Figure 7A). IκBα ubiquitination and proteasomal degradation were determined to be required for the activation of NF-κB. To investigate whether Rig-G impacts IκBα ubiquitination, we performed an immunoprecipitation assay using Tet-On A549 cells. Rig-G significantly impaired the ubiquitination of IκBα in Rig-G overexpression cells (Figure 7B). TNFα has been previously reported to trigger NF-κB activation [20]. The present study showed that although treatment of cells with TNFα increased the activation of NF-κB, as expected, Rig-G blocked the TNFα-mediated activation of NF-κB (Figure 7C). The NF-κB-driven luciferase reporter gene assay showed that TNFα-induced binding of NF-κB to its DNA consensus sequence was significantly decreased in Rig-G overexpressing cells, as displayed by a decrease in luciferase activation, thereby demonstrating the inhibitory effect of Rig-G on TNFα-mediated activation of NF-κB (Figure 7D). Furthermore, the cells overexpressing Rig-G revealed marked inhibition of TNFα-induced degradation of IκBα, as well as a significant decrease in ubiquitination (Figure 7E). To gain further insights into the role of NF-κB in RIG-G-induced cell growth inhibition, we generated stable A549 cell lines (pTRE and Tet-On Rig-G) containing short hairpin RNA (shRNAs) specific to p65 (Figure 8A). Cell proliferation was markedly reduced when NF-κB was inactivated in pTRE A549 cells (Figure 8B). NF-κB deficiency in pTRE A549 cells also decreased colony formation ability (Figure 8C, 8D). We observed a significant decrease in cell proliferation and colony formation in Tet-On Rig-G A549 cells with NF-κB deficiency in comparison with control (Tet-On Rig-G A549 cells) (Figure 8B, 8C, 8D). Collectively, these results indicated that the inhibition of NF-κB activation is likely the major cell proliferation-inhibiting mechanism of Rig-G in NSCLC cells.

### Rig-G inhibits NF-κB activity and is mediated by STAT3

**Figure 9:**
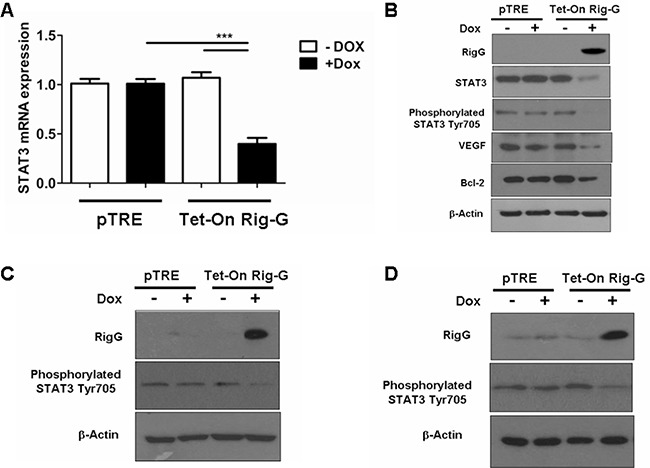
Rig-G inhibits STAT3 activity **A.** Real-time PCR analysis of STAT3 mRNA expression in A549 sublines pTRE and Tet-On Rig-G. The cells were respectively cultured in the presence or absence of Dox (2 μg/mL) for 24 h. The results are expressed as the mean ± SEM (n = 5), ***p < 0.001. **B.** A549 sublines pTRE and Tet-On Rig-G were analyzed for the expression of the indicated proteins by immunoblot analysis. **C** and **D.** H1792 (C), and Calu-1 (D) sublines pTRE and Tet-On Rig-G were analyzed for the expression of the indicated proteins by immunoblot analysis.

To gain further insights into the molecular mechanism by which Rig-G inhibits NF-κB activity, the expression of STAT3 and STAT3-related genes was analyzed before and after Rig-G induction. In agreement with the reduction in IL-6 production, we observed that the levels of STAT3 mRNA and protein were inhibited in Rig-G overexpressing A549 cells, but not in control cells (Figure 9A, 9B). STAT3 phosphorylation on Tyr705 was also significantly decreased in A549 cells with Rig-G overexpression (Figure 9B). Moreover, the expression of STAT3 targeting gene products, Bcl2 and VEGF, in A549 cells was also reduced by Rig-G (Figure 9B). The above experiments of STAT3 inactivation were repeated using the other ATRA-sensitive lung cancer cell lines, H1792, and Calu-1. Results were very similar to those obtained in A549 cell line with RIG-G overexpression. The expression of RIG-G resulted in considerable reduction in phosphorylated STAT3 in both cell lines (Figure 9C and 9D).

**Figure 10:**
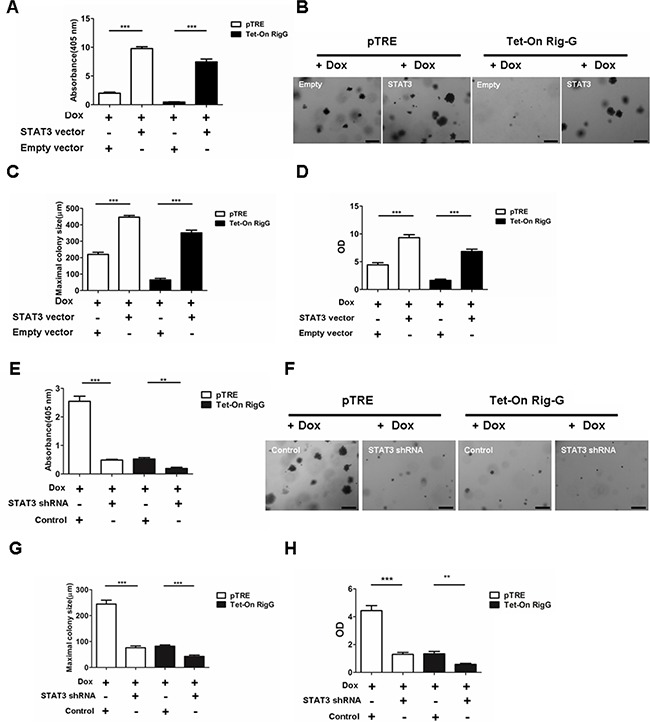
STAT3 inhibition is required for Rig-G decreasing lung cancer growth **A.**The cells were cultured in the presence of Dox (2 μg/mL). NF-κB activity (ELISA assay) in A549 sublines pTRE and Tet-On Rig-G respectively transfected with an empty vector or STAT3 vector. The results are expressed as the mean ± SEM (n = 3), ***p < 0.001. **B.** Colony formation assay of A549 sublines pTRE and Tet-On Rig-G respectively transfected with empty vector or STAT3 vector. Scale bars = 500 μm. **C.** The maximum colony size of the indicated cells on soft agar was determined. The results are expressed as the mean ± SEM, ***p < 0.001. **D.** The proliferation of the indicated cells was measured by ELISA (BrdU labeling) analysis. The results are expressed as the mean ± SEM, ***p < 0.001. **E.** A549 sublines pTRE and Tet-On Rig-G were transduced with indicated shRNA plasmids. After selection, the cells were cultured in the presence of Dox (2 μg/mL). NF-κB activity was assay by ELISA. The results are expressed as the mean ± SEM (n = 3), **p < 0.01; ***p < 0.001. **F.** The growth of tumor cells transduced with indicated shRNA plasmids in soft agar was assessed by using an anchorage-independent colony formation assay. Scale bars = 500 μm. **G.** The maximum colony size assay in A549 sublines pTRE and Tet-On Rig-G cells transfected with the indicated shRNA plasmids. The results are expressed as the mean ± SEM, ***p < 0.001. **H.** The indicated cells were transduced with control or STAT3 shRNA plasmids, and their proliferation was measured by ELISA (BrdU labeling) analysis. The results are expressed as the mean ± SEM, **p < 0.01; ***p < 0.001.

To confirm the effect of STAT3 restoration on NF-κB activity, we stably transfected Tet-On A549 cells with a STAT3-expression construct or an empty vector as control. As expected, STAT3 transfection restored NF-κB activity (Figure 10A), as well as antagonized Rig-G-induced inhibition of colony formation (Figure 10B, 10C) and growth (Figure 10D) in Tet-On A549 cells compared to that in control cells, which showed no Rig-G overexpression. Next, we generated stable A549 cell lines containing short hairpin RNA (shRNAs) specific to STAT3. Silencing of STAT3 significantly reduced NF-κB activity in pTRE A549 cells (Figure 10E). Furthermore, in A549 cells with Rig-G overexpression, silencing of STAT3 further reduced the activation of NF-κB (Figure 10E). We also examined the proliferation and colony formation ability of A549 cell with STAT3-knocked down. We found that silencing of STAT3 also reduced the proliferation and colony formation ability of A549 cell without Rig-G overexpression (Figure 10F, 10G, 10H). Silencing of STAT3 further inhibited colony formation and growth of A549 cell with Rig-G overexpression (Figure 10F, 10G, 10H). These findings suggested that STAT3 might be the main mediator through which Rig-G inhibits NF-κB in cancer cells.

**Figure 11:**
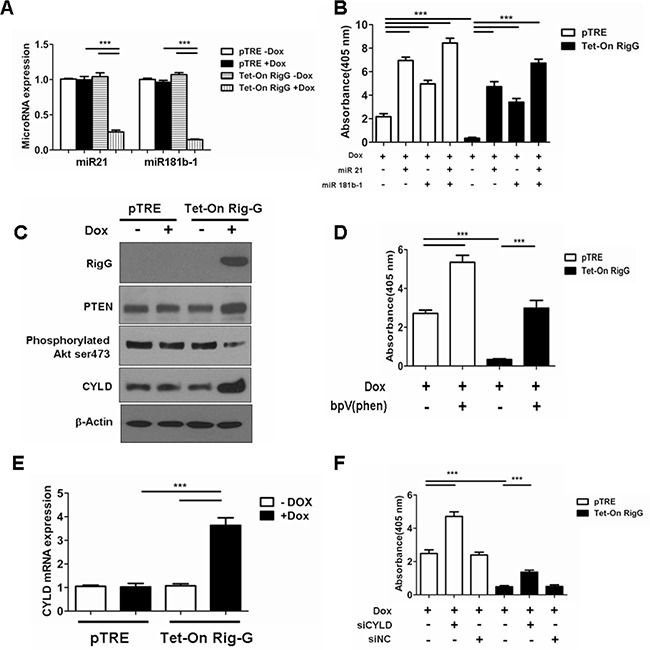
Rig-G inhibits NF-κB activation through downregulation of MiR21/PTEN/Akt and miR181b-1/CYLD pathways **A.** The cells were respectively cultured in the presence or absence of Dox (2 μg/mL) for 24 h. Real-time PCR analysis of MicroRNA expression levels in A549 cell lines with and without Rig-G overexpression. The results are expressed as the mean ± SEM (n = 5), ***p < 0.001. **B.** NF-κB activation assay of A549 sublines pTRE and Tet-On Rig-G treated with miR-21 (100 nM), miR-181b-1 (100 nM) for 24 h. The results are expressed as the mean ± SEM (n = 3), ***p < 0.001. **C.** A549 sublines pTRE and Tet-On Rig-G were analyzed for the expression of the indicated proteins by immunoblot analysis. **D.** NF-κB activation assay of A549 sublines pTRE and Tet-On Rig-G treated with PTEN inhibitor bpV (phen) (10 μM) for 24 h. The results are expressed as the mean ± SEM (n = 3), ***p < 0.001. **E.** Real-time PCR analysis of CYLD mRNA expression in A549 sublines pTRE and Tet-On Rig-G. The cells were respectively cultured in the presence or absence of Dox (2 μg/mL). The results are expressed as the mean ± SEM (n = 5), ***p < 0.001. **F.** NF-κB activity (ELISA assay) in A549 sublines pTRE and Tet-On Rig-G treated with siCYLD or siNC (negative control). The results are expressed as the mean ± SEM (n = 3), ***p < 0.001.

Previous studies have shown that STAT3 activation directly induces NF-κB-activated miR21 and miR181b-1 expression [17]. We propose that STAT3 and its downstream effectors are responsible for Rig-G-induced inhibition of NF-κB. As expected, we observed a dramatic decrease in the levels of miR21 and miR181b-1 expression in Rig-G-expressing A549 cells (Figure 11A), and the individual expression of miR21 or miR181b-1 significantly reduced the inhibition of NF-κB activity by Rig-G (Figure 11B), indicating that the downregulation of these microRNAs is important for Rig-G-mediated inhibition of NF-κB activation.

In accordance to a previous study that determined that the PTEN/Akt pathway that regulates NF-κB activity was controlled by miR21 in tumor cells, we examined PTEN expression and Akt phosphorylation in A549 cells before and after Rig-G induction. We observed that PTEN expression in cultured A549 cells was upregulated by Rig-G (Figure 11C). Akt phosphorylation of Ser473 was decreased in the total protein prepared from Rig-G overexpressing A549 cells (Figure 11C). Furthermore, treatment of cells with the PTEN inhibitor, bpV(phen) (10 μM), blocked the Rig-G-mediated inhibition of NF-κB activation (Figure 11D). These findings suggest that Rig-G inhibits NF-κB activity by regulating the PTEN/Akt pathway.

We have shown previously that Rig-G prevents the ubiquitination of IκBα, and CYLD, a deubiquitinating enzyme, is a direct target of miR181b-1 [17]. To address whether the observed inhibition of ubiquitination in IκBα is relevant to the expression of CYLD, we examined the expression of this enzyme. A significant upregulation of the CYLD was observed in the A549 cells with Rig-G overexpression both at the level of protein and mRNA (Figure 11C, 11E). Silencing of CYLD in A549 cells via siRNA significantly increased NF-κB activation of Rig-G overexpressing A549 cells compared to cells without Rig-G overexpression (Figure 11F). Thus, the miR181b-1/CYLD pathway is also involved in the inhibition of NF-κB activation subsequent to Rig-G expression.

**Figure 12:**
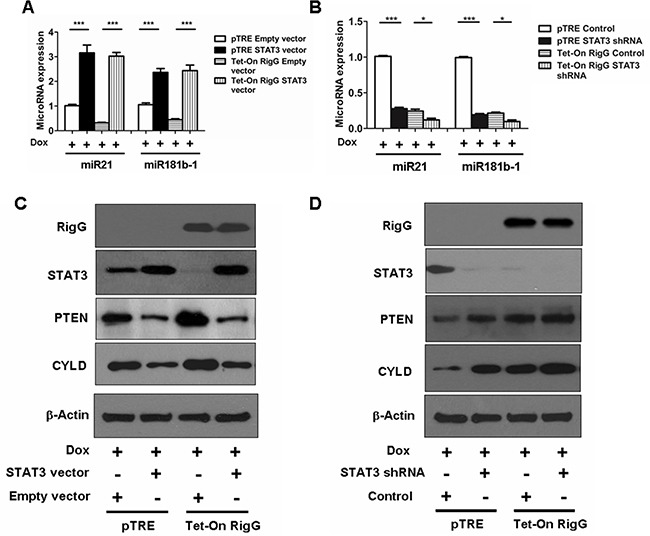
Rig-G downregulates MiR21/PTEN and miR181b-1/CYLD pathways through STAT3 **A** and **B.** MiR181b-1 and miR21 expression levels were analyzed in A549 sublines pTRE and Tet-On Rig-G respectively transfected with indicated expression plasmids (A) or shRNA plasmids (B). The cells were cultured in the presence of Dox (2 μg/mL). The results are expressed as the mean ± SEM (n = 5), ***p < 0.001. (B) A549 sublines pTRE and Tet-On Rig-G respectively transfected with indicated expression plasmids **C.** or shRNA plasmids **D.** were analyzed for the expression of the indicated proteins by immunoblot analysis.

To further confirm the role of STAT3, we analyzed the expression of STAT3 targeting genes, miR21, miR181b-1, CYLD and PTEN, in Rig-G regulated STAT3 expression or knocked down A549 cells. In Rig-G overexpressing A549 cells, Rig-G expression resulted in a reduction in the induction of miR21 and miR181b-1 and an increase in the expression of CYLD and PTEN, which is blocked by the ectopic expression of STAT3 (Figure 12A, 12C). Silencing of STAT3 in pTRE A549 cells significantly reduced the expression of miR21 (Figure 12B). In Rig-G overexpressing A549 cells, knockdown of STAT3 exhibited a lower miR21 level than control (Figure 12B). Similar results were seen in the expression of miR181b-1 (Figure 12B). Expression of CYLD and PTEN were induced in response to STAT3 knockdown or Rig-G overexpression, and both proteins has a modest increase in STAT3 knockdown A549 cells with Rig-G overexpression (Figure 12D). These results are consistent with those obtained by measuring miR21 and miR181b-1.

Taken together, these results are consistent with a pathway in which Rig-G inhibits STAT3, leading to decreased miR21 and miR181b-1 expression and subsequent regulation of PTEN/Akt and CYLD/IκBα pathways, which inhibit the activity of NF-κB.

## DISCUSSION

The main finding of the present study is that Rig-G is a growth inhibition factor of lung cancer cells. The protein inhibits NF-κB activity by suppressing STAT3 in lung cancer cells. The decreased expression of miR21 and miR181b-1 and subsequent modulation of PTEN/Akt and CYLD/IκBα signaling axis mediate the STAT3-NF-κB pathway via Rig-G.

It has been previously reported that the induction of Rig-G by ATRA occurs in various types of cancers, not merely in acute promyelocytic leukemia but also in head and neck squamous carcinoma cells and ovarian cancer, suggesting that Rig-G may have similar effects in different cell contexts [8,18]. In the present study, we examined the expression of Rig-G in lung cancer cell lines. We found that lung cancer cell lines such as A549, H1792, and Calu-1 exhibited ATRA-induced expression of Rig-G. The baseline expression of Rig-G varied in different lung cancer cell lines, although a rapid induction was observed at 24 h after ATRA treatment, which exhibited ATRA sensitivity that was comparable to some lung cancer cells such as Calu-1 and H1792. The expression regulation of Rig-G may have resulted from different mechanisms: (1) Our previous studies showed that ATRA induces Rig-G expression by activation of the JAK-STAT1 pathway, which is involved in the expression of IRF-1/IFN-α [8]. (2) IRF-9/STAT2 functional interaction drives the expression of Rig-G independent of STAT1 and the classical JAK-STAT pathway [21]. IRF-1 also induces Rig-G expression via an IRF-9/STAT2-dependent or IRF-9/STAT2-independent mechanism [21]. (3) STAT1 could significantly enhance the effects of the IRF-9/STAT2 complex or IRF-1 on Rig-G induction via an activated JAK-STAT pathway [22]. (4) The expression of PU.1 by ATRA directly binds to the promoter and increases the expression of Rig-G [23].

Although Rig-G is known as an anti-proliferative factor, it has only been proven to be critical for growth inhibition in a leukemia model [8]. Here, we demonstrate that specific Rig-G overexpression in lung cancer cells interferes with tumor cell proliferation and tumor growth in xenograft models. Based on the expression of Rig-G in ATRA-sensitive cells (Calu-1 and H1792) or ATRA-resistant cells (A549), a correlation between the ability of ATRA to inhibit lung cancer cell growth and ATRA-induced expression level of Rig-G was detected. Moreover, the relatively high level of Rig-G baseline expression and higher level of ATRA-induced Rig-G expression are highly likely an important mechanisms underlying the growth inhibitory effects of ATRA. Based on these results, we performed growth inhibitory experiments using a Rig-G overexpression cell model. Our results showed that Rig-G overexpression suppresses the growth of lung cancer cell lines, including ATRA-resistant A549 cells, indicating that Rig-G expression is necessary but not sufficient for conferring ATRA sensitivity to cells. Thus, because of the strong effect of Rig-G in inhibiting growth and colony formation in the ATRA-resistant cell line A549 cell, we choose it to perform the subsequent experiments for demonstrating the tumor inhibiting effect of Rig-G in the lung cancer.

The development of lung cancer has been related to inflammation, cell proliferation, apoptosis, and various signaling pathways, including the NF-κB signaling pathways [24,25]. NF-κB controls the expression of anti-apoptotic, pro-proliferative, and immune response genes, which is required in the pathogenesis of various cancers, including lung cancer, colon cancer, and hepatocellular carcinoma [26, 27]. Our previous studies have demonstrated that Rig-G upregulates the expression of cell cycle inhibitors p21 and p27 by downregulating c-myc or by preventing p27 from JAB-1-dependent and ubiquitin/proteasome-mediated degradation [8]. In the present study, the tumor-inhibiting activity of Rig-G is executed by antagonizing NF-κB activation. NF-κB in lung cancer cells is responsible for the induction of several anti-apoptotic and pro-proliferative proteins, as well as inflammatory factors, including Bcl-XL, Bcl-2, PCNA, and IL-6 [24, 28]. The inhibition of NF-κB by Rig-G appears to be an important factor for antitumor activity as only lung cancer cell growth during Rig-G overexpression showed a low rate.

The collaboration and crosstalk of STAT3 and NF-κB play pivotal roles in the pathogenesis of various cancers [12,13]. There are several interactions and forms of crosstalk between NF-κB and STAT3: (1) NF-κB physically interacts with STAT3 [16, 29]; (2) these factors cooperate with gene promoters/enhancers [30]; (3) the cooperation of STAT3 and NF-κB builds a positive feedback loop in its activation process [17]. In lung cancer, constitutively activated STAT3 has been observed in neoplastic cells [31–33]. In our case, we have provided evidence that the inhibition of lung cancer growth of the overexpressing Rig-G line was blocked by the transgenic expression of STAT3, indicating that the function of Rig-G in tumor inhibition involves STAT3. MiR21 and miR181b-1 are important STAT3 effectors whose ablation in A549 cells results in decreased tumor growth [17]. Here, we demonstrate that specific Rig-G overexpression in lung cancer cells interferes with the expression of STAT3, miR21, and miR181b-1. A study conducted by IIiopoulos et al. suggested a mechanism in which STAT3 activation of MiR21 and miR181b-1 is required to activate the NF-κB pathway via PTEN/Akt and CYLD in an ER-Src model of oncogenesis [17]. In the current study, Rig-G inhibits NF-κB activity by increasing PTEN expression in lung cancer cells. In turn, Rig-G activated CYLD by inhibiting miR181b-1, and subsequently, NF-κB. Additionally, NF-κB inhibition resulted in a decreased induction of IL-6, which is an important STAT3 activator that promotes tumor development and growth in colon cancer and lung cancer [34]. Thus, the overexpression of Rig-G in lung cancer cells builds a negative feedback loop during the development of tumor growth inhibition effect.

In conclusion, the results of the present study show that Rig-G inhibits lung cancer cell growth via MiR21/PTEN/Akt and miR181b-1/CYLD-dependent inhibition of NF-κB, which is associated with decreased STAT3 activity. Our findings contribute to a better understanding of the antitumor effect mechanism of Rig-G, as well as offer a novel strategy for lung cancer therapy.

## MATERIALS AND METHODS

### Cell culture

Human NSCLC cell lines A549, H1792, and Calu-1 (all three from ATCC) were cultured in Dulbeccos's modified Eagle's medium (DMEM) (Hyclone Laboratories, Inc., South, UT, USA) supplemented with 10% fetal calf serum (FCS) (Invitrogen, Grand Island, NY, USA), 100 U/mL penicillin and 100 U/mL streptomycin (Hyclone Laboratories., Inc.). Cell cultures were performed at 37°C in humidified air with 5% CO_2_. ATRA (1 μM, Sigma-Aldrich, St. Louis, MO, USA) was applied to the cells for 96 h.

### Transfection and establishment of lung cancer Tet-On-Rig-G stable transformants

Rig-G cDNA (1.5 kb in size) was constructed in an expression vector pTRE by using the Tet-On system (Clontech Laboratories, Mountain View, CA, USA) as described elsewhere [8]. The lung cancer Tet-On cells (A549, H1792, and Calu-1 cells) were stably transfected with a pTet-On regulatory vector by using an electroporation method. Positive clones were selected by 800 μg/mL G418 (Clontech Laboratories). After establishing the lung cancer Tet-On stable cells, these were transfected again with pTRE-Rig-G. Electroporation was performed using a Gene-Pulser (Bio-Rad) at 280 V and 960 mF in 0.4-cm cuvettes (Bio-Rad, Hercules, CA, USA). Individual positive clones were then isolated in the growth medium (containing 200 μg/mL hygromycin B) via limiting dilution analysis in 96-well plates. In the presence of Dox (2 μg/mL, Sigma-Aldrich) for 24 h, the lung cancer Tet-On-Rig-G cell lines (A549 Tet-On-Rig-G, H1792 Tet-On-Rig-G, and Calu-1 Tet-On-Rig-G) expressed Rig-G. An empty vector pTRE was used as a control. For TNFα (Invitrogen) stimulation, after DOX treatment for 24 h, Tet-On-Rig-G cells and pTRE cells were treated with 1 ng/mL TNFα for 1 h. For establishment of the STAT3 expression or knockdown stable cell line, A549 Tet-On-Rig-G cells, and A549 pTRE, cells were transfected with STAT3 plasmids, STAT3 shRNA plasmids or empty vectors (all from OriGene Technologies, Inc. Rockville, MD, USA), respectively, by electroporation. Individual stable cells were selected under 800 μg/mL G418 (Clontech Laboratories) or 5 μg/mL puromycin (Invitrogen). RNA interference (RNAi) plasmids Rig-G were constructed with the following sequences for the Rig-G: 5′-CACCGACAATCCCATCAGCGC TACTT CAAGAGAGTAGCGCTGATGGGATTG-3′ and 5′-AAA ACAATCCCATC AGCGCTACTCTCTTGAAGTAGCGCTGATGGGATTGTC-3′. Puromycin was added at a concentration of 5 μg/ml. NF-κB p65 RNAi plasmids were described [24].

### Western blot analysis

Western blot analysis was performed as described elsewhere [35]. Briefly, 30mg total protein or 5mg nuclear protein were loaded on 10% SDS polyacrylamide gels, electrophoresed, and blotted onto Hybond-C Extra membranes (Amersham Bioscience, Buckinghamshire, UK). Primary antibodies used for western blot analysis included the following: rabbit anti Rig-G (a gift from Dr. Jianhua Tong [7]), mouse anti-cyclin D1, mouse anti-PCNA, mouse anti-p21, mouse anti-STAT3, rabbit anti- Stat3 (pTyr705) rabbit anti-c-myc, rabbit anti-Akt (pSer473), rabbit anti-PTEN, rabbit anti- IκBα, rabbit anti-CYLD, rabbit anti-Bcl-2 (all eleven from Cell Signaling Technology), mouse anti-ubiquitin, rabbit anti-p27, rabbit anti-p65, rabbit anti-LaminB (all four from Santa Cruz Biotechnology, Santa Cruz, CA, USA), rabbit anti-VEGF (from Abcam, Cambridge, UK), and mouse anti-β-actin (Sigma Aldrich). HRP-conjugated goat anti-rabbit (Santa Cruz) or rabbit anti-mouse (Dako, Carpinteria, CA, USA) was used as secondary antibody.

For co-immunoprecipitation, cells were incubated with the proteasome inhibitor MG132 (20μM, Cell Signaling Technology) for 5h. Protein extracts were prepared in a buffer containing 150 mM NaCl, 50 mM Tris-HCl (pH 8.0), and 0.5% Non-idet P-40, and then mixed with protein A-agarose (Santa Cruz Biotechnology) and rabbit anti-IκBα at 4°C overnight with continuous rotation. The precipitated proteins were eluted by boiling beads in an SDS-loading buffer and analyzed by western blotting.

### RNA isolation and real-time PCR analysis

Total RNAs were isolated from cells using an RNeasy plus mini kit (Qiagen, Santa Clarita, CA, USA), following the manufacturer's recommendations. Real-time PCR reaction mixtures have been previously described [36]. Briefly, cDNA was synthesized by reverse transcription using a First-Strand cDNA Synthesis kit (Invitrogen). Real-time PCR was performed using the QPCR SYBR Green Mix (Bio-Rad) on an AB 7300 Real time PCR system machine (AB Applied Biosystems, Singapore). The following PCR primers were used: β-actin, 5′-AGCCTCGCCTTT GCCGA-3′ and 5′-CTGGTGCCTGGGGCG-3′; Rig-G, 5′-GAAGAAATGAAAGG GCGAAGG-3′ and 5′-AGG ACATCTGTTTGGCAAGGAG-3′; STAT3, 5′-CCAG TCAGTGACCAGGCAGAAG-3′ and 5′-GCACGTACT CCATCGCTGACA-3′; CYLD, 5′-TCTATGGGGTAATC CGTTGG-3′ and 5′- CAGCCTGCACACTCATCT TC-3′; TNFα, 5′-CCCAGGCAGTCAGATCATCTTC-3′ and 5′-AGCTGCCCCTCA GCTTGA-3′; IL-6, 5′-TCAC CAGGCAAGTCTCCTCATTG -3′ and 5′-ACTC CTTC TCCACAAGCGCCTT-3′; IL-1β, 5′-TCTGA ATTCTATGGCAGAAGTACCTGA GC-3′ and 5′-TTC GGATCCGGAAGACACAAATTGC-3′; miR-181b-1, 5′-ACACT CCAGCTGGGAACATTCATTGCTGTC GG-3′ and 5′- CTCAACTGGTGTCGTGG A-3′; miR-21, 5′-GCCGCTAGCTTATCAGACTGATGT-3′ and 5′-GTGCAGGGTC CGAGGT-3′. Specificity of RT-PCR was controlled by ‘no reverse transcription’ controls and melting curve analysis. Quantitative PCR results were obtained using the DDCT (cycle threshold) method. Data were normalized to the β-actin levels in each sample.

### BrdU ELISA cell proliferation assay

Lung cancer cell proliferation was determined using a commercially available cell proliferation ELISA, BrdU (colorimetric) kit (Roche, Mannheim, Germany), as previously described [35]. For ATRA stimulation, the cells were stimulated with 1 μM ATRA for 96 h. For Rig-G overexpression, after 5 days of Dox treatment, tumor cell supernatants were aspirated, and growth media that contained 10 μM BrdU was added. Tumor cells were incubated for an additional 2 h at 37°C, after which the rate of cell proliferation was measured.

### Anchorage-independent colony formation assay

A colony formation assay was performed as described elsewhere [35]. Briefly, a bottom layer consisting of 0.5% (w/v) agar in 0.8 mL of DMEM supplemented with 10% (v/v) FBS was first allowed to solidify in each well. Cell lines were suspended in 0.6 mL 0.3% (w/v) agar containing 10% (v/v) FBS and plated onto the bottom layer. ATRA (1 μM) or Dox (2 μg/mL) were used at the indicated concentrations in the media. Colony forming efficiency was determined 2 to 3 weeks after plating and cultivation in a humidified 5% CO2 atmosphere at 37°C. The colonies were visualized after staining with 0.005% crystal violet.

### Tumor xenograft models

Female BALB/c-nu/nu mice were obtained from the National Rodent Laboratory Animal Resource (Shanghai Branch, PRC) and maintained at the pathogen-free Central Animal Facility of the Tongji Hospital of Tongji University. This study was conducted in strict accordance with the recommendations depicted in the Guidelines for the Care and Use of Laboratory Animals of the National Institutes of Health in Bethesda, MD, USA. All animal experiments were approved by the Tongji Hospital of Tongji University Ethics Committee on the Use and Care of Animals. All surgery was performed under sodium pentobarbital anesthesia, and all efforts were made to minimize suffering.

A549 Tet-On-Rig-G cells or A549 pTRE cells (density: 1 × 10^6^) were injected subcutaneously in the right flank of nude mice. Tumor growth was monitored every 5 days, and tumor volumes were calculated by using the equation: V(mm^3^) = a × b^2^/2, where a is the largest diameter and b is the perpendicular diameter [15]. When the tumors reached a size of 70 mm^3^, mice were fed Dox (1 μg/mL mixed in drinking water) or water to regulate Rig-G expression in mice xenografts. At the end of the experiments (40 days), the mice were euthanized, and tumors were resected and fixed in 3.7% buffered formalin.

### Immunohistochemical analyses

Immunohistochemistry was performed as described elsewhere [37]. The following primary antibodies were used for immunohistochemical analysis: mouse anti-Ki-67 (Abcam, Cambridge, UK) and rabbit anti Rig-G (a gift from Dr. Jianhua Tong [7]). Secondary antibody incubation and staining were performed using the EnVision®+ System–HRP (DAB) kit (Dako), according to the manufacturer's recommendations. TUNEL staining was performed using the DeadEnd Colorimetric TUNEL System kit (Promega, Madison, WI, USA). The number of Ki-67-positive tumor cells and the total number of tumor cells were counted in six microscopic fields of a randomly selected tumor, and the mean value was calculated as the percentage of Ki-67-positive tumor cells.

### mRNA expression array

Total RNA was isolated from A549 Tet-On-Rig-G cells or A549 pTRE cells using an RNeasy Mini kit (Qiagen, Hilden, Germany) according to the manufacturer's recommendation. Total mRNA was labeled with Cy5 and then hybridized to Human Whole Genome OneArray^TM^ Version 6.1 (Phalanx Biotech Group, Xinzhu, Taiwan), scanned with an Axon 4000 scanner (Molecular Devices, Sunnyvale, CA, USA). Spot quantification was done with the use of genepix 4.1 software. Hierarchical clustering of the expression profiles was analyzed by Cluster 3.0 (Molecular Devices). For each group, three samples were analyzed. Differentially expressed genes with fold changes of >2.0 were applied to pathway enrichment analysis. GSEA were performed for all KEGG [37] using the GeneTrail server [38].

### ELISA assay for NF-κB activation

NF-κB activation in cells were analyzed by using TransAM® NF-κB/p65 ELISA kits (Active motif, Carlsbad, CA, USA), according to the manufacturer's instructions. For PTEN inhibition, PTEN inhibitor bpV(phen) (Cell Signaling Technology, Danvers, MA, USA) was added at a concentration of 10 μM. For microRNA stimulation, A549 Tet-On-Rig-G cells or A549 pTRE cells were treated with 100 μM of microRNAs (miR21 or miR181b-1) for 24 h, whereupon Dox (2 μg/mL) was added and NF-κB activation was assessed 24 h later (total time: 48 h).

### siRNA experiments

A549 Tet-On-Rig-G cells or A549 pTRE cells were transfected with siRNAs (100 nM) from Ambion (Austin, TX, USA) against CYLD using siPORT NeoFX transfection agent (Invitrogen). Approximately 100 nM of siRNA (Ambion Inc.) was used as control. Approximately 24 h after transfection, the resulting cells were treated with Dox (2 mg/mL), and NF-κB activation was assessed 24 h later (total time: 48 h).

### Luciferase reporter assay

The NF-κB luciferase adenovirus plasmid pNF-κB-Luc (Clontech) containing multiple copies of the NF-κB consensus sequence was used to monitor NF-κB activation [39]. A549 Tet-On-Rig-G cells or A549 pTRE cells were infected with a luciferase adenovirus plasmid pNF-κB-Luc. After 24 h of incubation, the infected cells were treated with Dox (2 μg/mL) for 24 h, and then subsequently stimulated with TNFα (1 μg/μL) for 1 h and then lysed, and luciferase reporter gene activity was determined by using the luciferase reporter assay kit (Promega, Madison, WI, USA).

### Immunofluorescence staining

The intracellular localization of proteins was analyzed directly on culture coverslips. The adherent cells grown on coverslips were washed in PBS and fixed in methanol at −20°C for 10 min, then washed in PBS. After drying overnight, the cells were fixed in acetone at 4°C for 10 min and air-dried. The indicated primary antibodies were diluted in PBS and incubated with the permeabilized cells for 1-2 h. Subsequently, secondary antibodies, including Alexa Fluor® 488-conjugated anti-rabbit and Alexa Fluor® 594-conjugated anti-rabbit antibody (Cell Signaling Technology), were incubated with cells for 1 h. All incubations were carried out at room temperature and followed by three washes in PBS. Finally, the slides were mounted with 5 μl mounting medium containing DAPI (Vector Laboratories, Burlingame, CA) and examined by microscopy (Olympus BX60, Japan) with CCD camera (Model 4.2, Diagnostic, USA).

### Electrophoretic mobility shift assay (EMSA)

EMSA were performed using the LightShift Chemiluminescent EMSA Kit (Thermo Scientific, Rockford, Ll, USA). Briefly, nuclear proteins were isolated from lung tumor cells using Nuclear Extract Kit (Active Motif, Carlsbad, CA, USA) and NF-κB DNA binding activity was measured by EMSA. Nuclear proteins were incubated with biotin-labeled double-stranded NF-κB consensus probe at room temperature for 30 min. DNA–protein complexes were resolved on a 5% nondenaturing polyacrylamide gel prepared in 45 mM Tris-borate and 1 mM EDTA (TBE) buffer. The specimens were electrotransferred onto a 0.4-μm Biodyne B nylon membrane (Thermo Scientific) at 380 mA for 30 min at 4 °C. The membrane was UV cross-linked and binding activity of NF-κB to the probe was determined using a chemiluminescent EMSA kit. For EMSA supershift experiments, nuclear extract were incubated for 30 min at room temperature with 2 mg of rabbit anti-p65 antibody (Santa Cruz) before addition of labeled oligonucleotides.

### Statistical analysis

Data are expressed as the mean±SEM. Comparisons between groups were analyzed by using the *t*-test (two-sided). A P value of < 0.05 was considered statistically significant.
